# Excessive daytime sleepiness in a model of Parkinson’s disease improved by low-frequency stimulation of the pedunculopontine nucleus

**DOI:** 10.1038/s41531-023-00455-7

**Published:** 2023-01-25

**Authors:** Aurélie Davin, Stéphan Chabardès, Annaelle Devergnas, Caroline Benstaali, Claire-Anne N. Gutekunst, Olivier David, Napoléon Torres-Martinez, Brigitte Piallat

**Affiliations:** 1 Univ. Grenoble Alpes, CEA, LETI, Clinatec, 38000 Grenoble, France; 2 Univ. Grenoble Alpes, Inserm, U1216, Grenoble Institut Neurosciences, 38000 Grenoble, France; 3 Univ. Grenoble Alpes, Inserm, Department of Neurosurgery, 38000 Grenoble, France; 4 Yerkes National Primate Research Center, 30307 Atlanta, USA; 5 Emory University School of Medicine, 30307 Atlanta, GA USA; 6 Univ. Aix Marseille, Inserm, INS, Institut de Neurosciences des Systèmes, 13000 Marseille, France

**Keywords:** Circadian rhythms and sleep, Parkinson's disease

## Abstract

Patients with Parkinson’s disease often complain of excessive daytime sleepiness which negatively impacts their quality of life. The pedunculopontine nucleus, proposed as a target for deep brain stimulation to improve freezing of gait in Parkinson’s disease, is also known to play a key role in the arousal system. Thus, the putative control of excessive daytime sleepiness by pedunculopontine nucleus area stimulation merits exploration for treating Parkinson’s disease patients. To this end, two adult nonhuman primates (*macaca fascicularis*) received a deep brain stimulation electrode implanted into the pedunculopontine nucleus area along with a polysomnographic equipment. Stimulation at low frequencies and high frequencies was studied, in healthy and then MPTP-treated nonhuman primates. Here, we observed that MPTP-treated nonhuman primates suffered from excessive daytime sleepiness and that low-frequency stimulation of the pedunculopontine nucleus area was effective in reducing daytime sleepiness. Indeed, low-frequency stimulation of the pedunculopontine nucleus area induced a significant increase in sleep onset latency, longer continuous periods of wakefulness and thus, a partially restored daytime wake architecture. These findings may contribute to the development of new therapeutic strategies in patients suffering from excessive daytime sleepiness.

## Introduction

Excessive daytime sleepiness (EDS) refers to sleepiness that occurs in situations when an individual would be expected to be awake and alert preventing the performance of daily activity^1,2^. EDS is a chronic problem that affects 5–12% of the population and 15–51% of patients suffering from Parkinson’s disease (PD)^3–5^ seriously impacting their quality of life^6,7^. The neurological substrates of sleepiness are not fully understood but could result from a variety of multifactorial causes including inadequate nighttime sleep quality, a circadian rhythm disorder, or a sedative effect of medications^8^. Sleepiness may also result from distinct neural systems that promote sleep or may reflect the waning of processes that maintain wakefulness. Numerous brain areas participate in the initiation and maintenance of alertness, known as part as the arousal systems, that are likely involved in EDS. The arousal systems are now clearly identified. The first pathway includes the upper brainstem nuclei^9^ (including the raphe nucleus, the locus coeruleus, the ventral periaqueductal gray matter and the pedunculopontine nucleus (PPN)), the hypothalamus^10,11^ (with orexin and histamine neurons) and the basal forebrain^11,12^. These different structures are highly interconnected^13^ and directly activate the cortex^11,14^. Among these different structures, the PPN was hypothesized relatively early to promote cortical desynchronization^15,16^ and belongs to another pathway of arousal, which does not directly activate the cortex but switches the thalamus from a synchronized to a desynchronized mode^17^, which is one of the criteria of arousal. The involvement of the PPN in promoting wakefulness is illustrated by its high neuronal activity during active wake and the decrease of this activity during non-REM sleep^18–20^. Moreover, the existence of a heterogeneous neuronal population within the PPN indicates that this nucleus an important participant in wakefulness. Indeed, the PPN is mainly comprised of cholinergic neurons^21^ as well as glutamatergic^22^ and GABAergic neurons^23^. Rodent studies have suggested that these varied neuronal populations are essential for maintaining global wakefulness^19,24,25^. Selective activation of the glutamatergic neurons induces prolonged wakefulness, activation of the cholinergic neurons suppresses low-frequency cortical activity during non-REM sleep and activation of the GABAergic neurons slightly reduces REM sleep^26^. During the last three decades, preclinical trials have highlighted the role of the PPN in locomotion^27,28^, making this nucleus a potential surgical target for deep brain stimulation (DBS) to treat freezing of gait in PD patients. Interestingly, our team has further established the involvement of the PPN in the control of wake/sleep behavior in PD patients, as we previously found that acute low-frequency stimulation (LFS; 25 and 10 Hz) of the PPN area increased wakefulness, whereas acute high-frequency stimulation (HFS; 80 Hz) of the PPN area triggered rapid-onset episodes of non-REM sleep^29^. These clinical observations reinforce the idea that wake/sleep behavior can be modulated by chronic PPN area stimulation. Further demonstration of wakefulness due to PPN area stimulation, with repeated evaluations during acute and chronic stimulation, in healthy and parkinsonian NHPs would allow the development of innovative DBS applications to reduce EDS in humans. The MPTP-treated NHP model is known to mimic both motor symptoms and nonmotor symptoms of PD^30–32^. In a previous paper, we fully characterized the sleep/wake disturbances in this model^33^, demonstrating its usefulness to evaluate the effect of target and parameters settings of a novel DBS method on EDS. For this purpose, the NHPs were equipped with a telemetric device to monitor the different wake and sleep stages as well as a DBS lead connected to an implanted stimulator in the PPN area. In the present study, we found that PPN-LFS was more effective than PPN-HFS for enhancing daytime wakefulness. Indeed, we showed that PPN-LFS induced significant increase in sleep onset latency in the morning, longer continuous periods of wakefulness and a decrease in daytime sleep time; tending towards a restored daytime wake architecture.

## Results

### Location of electrodes and PPN stimulation parameters

**Fig. 1:**
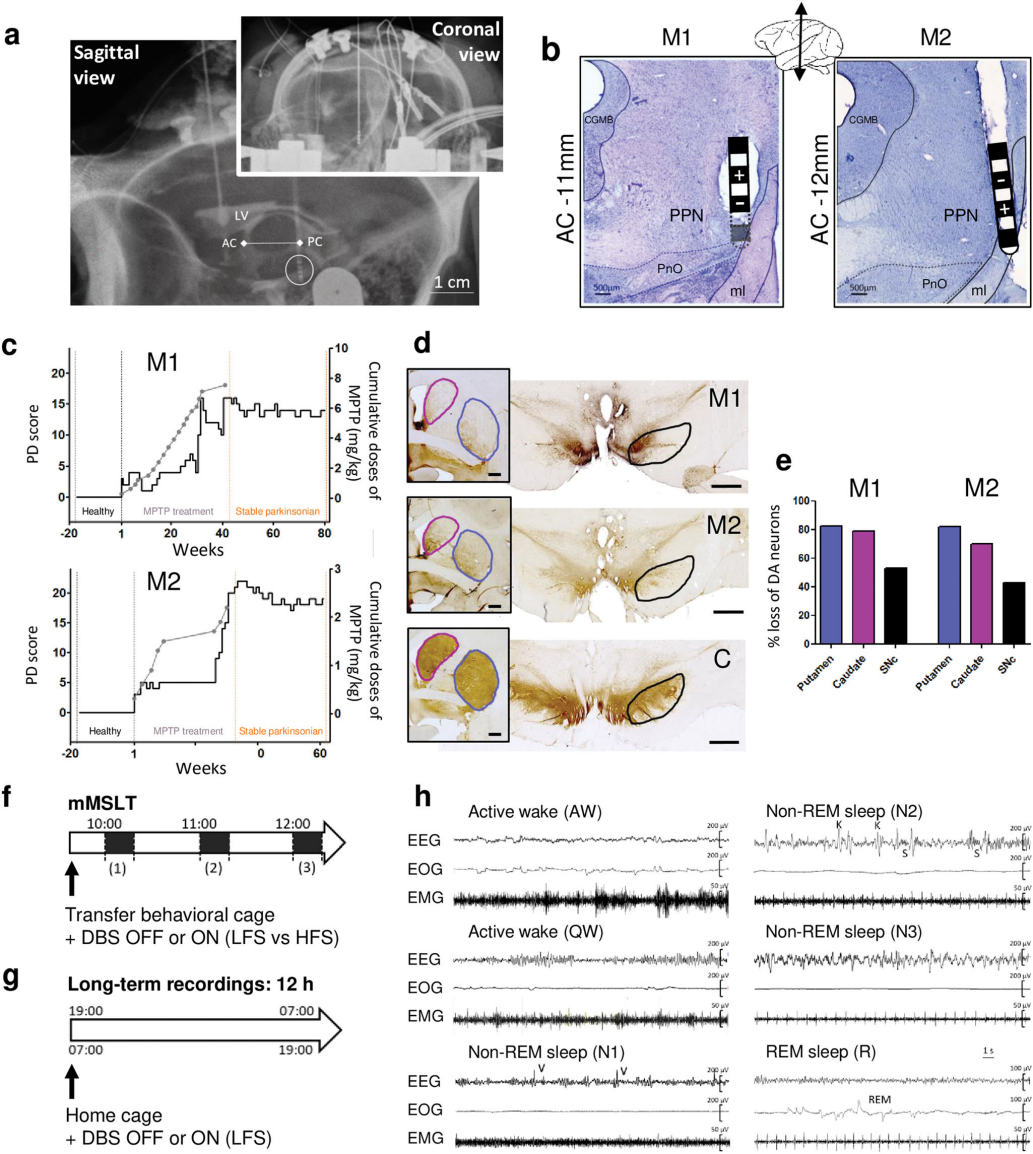
Surgery report, PD scores and experimental design. **a** X-ray of the final implantation of the electrode in the PPN area for M1, in both coronal and sagittal view with the internal landmarks AC-PC line determined by ventriculography (LV: lateral ventricle). **b** Micrographs of Cresyl-violet stained sections through the PPN of M1 and M2 showing postmortem reconstruction of the electrode track and position of the contacts, (-) is the active contact. CGMB = central gray substance of midbrain; PnO= oral pontine reticular nucleus; ml= medial lemniscus. **c** Graphs illustrating the longitudinal progression of parkinsonian syndrome for M1 and M2, induced by injection of chronic low doses of MPTP, based on weekly observations. The solid black line shows the PD score and the gray dotted line represents the cumulative dose of MPTP with each dot corresponding to an injection of 0.2–0.5 mg/kg. Note 3 key periods: the healthy period (black), the MPTP treatment period (gray) and the stable parkinsonian period (orange). During the healthy period the PD scores were 0/25. During the stable parkinsonian period the score was 13.8 ± 0.2 / 25 for M1 and 18.9 ± 0.1 / 25 for M2. **d** Micrographs showing tyrosine hydroxylase (TH) immunostaining, at the level of the striatum (framed image, scale bar= 2000µm) and the substantia nigra (scale bar= 2000µm) for M1, M2 and the control animal (C). The circled areas correspond to the striatal structures (putamen in blue and caudate nucleus in purple) and the substantia nigra in black. **e** Graphs showing the percent loss of TH expression in the putamen, the caudate nucleus and the substantia nigra of M1 and M2 compared to the control animal. Percent loss are calculated based on optical density method using the region of interest outlined in d. **f** Design of the modified multiple sleep latency test (mMSLT) with 20 min light-OFF at 10:00 h (1), 11:00 h (2) and 12:00 h (3), performed in behavioral cage and under different conditions: healthy or stable parkinsonian states with PPN-DBS OFF or ON (LFS vs. HFS). **g** Design of long-term recordings of 12 h nighttime (from 19:00 h to 7:00 h) and daytime (from 7:00 h to 19:00 h), performed in home cage and under different conditions: healthy or stable parkinsonian states with PPN-DBS OFF or ON (LFS only). **h** Polysomnographic recordings for wake/sleep stages analysis. Thirty seconds epochs showing active wake (AW), quiet wake (QW), non-REM sleep stage 1 (N1), stage 2 (N2), stage 3 (N3) and REM sleep (R).

The location of the implanted electrodes was guided during the surgery by ventriculography and verified postoperatively by radiography (Fig. 1a). To further evaluate the correct position of the electrode in the PPN area, we then applied a range of stimulation parameters studying the acute behavioral effects of PPN area stimulation on muscle tone and level of attention. These stimulation sweep tests showed that LFS (<30 Hz) induced hypertonic and alert behavior whereas HFS (>60 Hz) induced hypotonic behavior accompanied by yawns. Two stimulation frequencies were chosen (LFS = 20 Hz and HFS = 80 Hz) because they induced the most marked effects. The voltage was defined according to the threshold at which side effects appeared (contralateral myoclonus for M1 and contralateral hemiface contraction and nausea for M2) and set at 80% of this threshold (LFS and HFS = 5.6 V for M1, LFS = 3 V and HFS = 1.8 V for M2). Finally, the precise location of electrodes was confirmed postmortem showing implantation in the lateral part of the PPN in both animals, closer to the medial lemniscus for M2 (Fig. 1b).

### Motor scores and degree of dopamine depletion

Throughout the course of MPTP treatment, animals exhibited a gradual onset of motor symptoms and finally a stabilization of these symptoms, above a score of 10, which is considered a parkinsonian state, for a minimum of 25 weeks without recovery (PD score for M1: 13.8 ± 0.2 and for M2: 18.9 ± 0.1) (Fig. 1c). These motor symptoms included bradykinesia, posture disorder, important action tremors and a decrease in general activity. Neither LFS nor HFS of the PPN area significantly improved the general locomotion evaluated by the parkinsonian scale as well as actimetry (Supplementary Fig. 1a). Gait, posture or tremors were also not affected by stimulation. However, an increase in interactions with enrichments, such as poplar strips, pieces of rope, chewing rubber ball was observed with PPN-LFS in both animals (Supplementary Fig. 2b); behaviors more commonly associated with interested arousal than locomotor arousal. Postmortem analysis revealed a large decrease of TH expression in the striatum and substantia nigra of both MPTP-treated animals (M1 and M2) compared to the untreated control animal (C) (Fig. 1d), with approximately 80% loss at the level of the striatum (the putamen and caudate nucleus) and approximately 40% at the level of the substantia nigra (Fig. 1e).

### Excessive daytime sleepiness

**Fig. 2:**
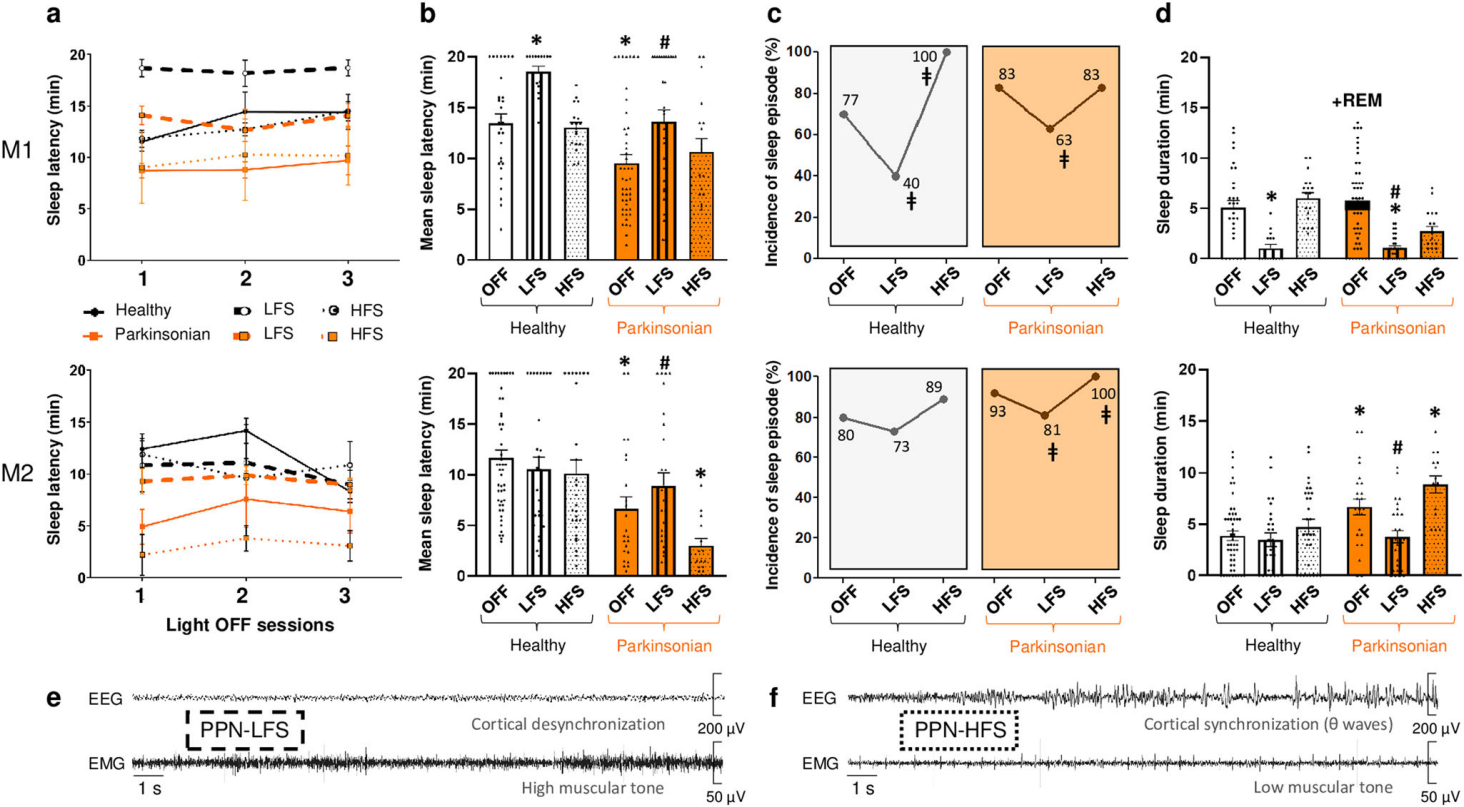
Panel of different sleep parameters obtained from mMSLTs. Sleep parameters were obtained in light-OFF sessions for each animal M1 and M2, OFF-stimulation versus ON-stimulation conditions in healthy (white) and parkinsonian (orange) states with low-frequency stimulation (LFS, vertical lines) and high-frequency stimulation (HFS, dots). **a** Sleep latency, expressed in minutes (mean ± SEM), for each light-OFF session (1) from 10:00 h to 10:20 h, (2) from 11:00 h to 11:20 h, (3) from 12:00 to 12:20 h for healthy (black line) and parkinsonian (orange line) states with LFS (bold dotted line) and HFS (thin dotted line), **b** Mean time of sleep latency, expressed in minutes (mean ± SEM), for all light-OFF sessions pooled together by condition, *p* < 0.05 * different from healthy OFF-stimulation; # different from parkinsonian OFF-stimulation: Kruskal–Wallis test followed by Dunn’s multiple comparisons test. **c** Occurren**c**e of at least 30 seconds of sleep during all light-OFF sessions, expressed in %, *p* < *0.05* ǂ different from OFF-stimulation: Fisher’s exact test. **d** Mean time of sleep duration, expressed in minutes (mean ± SEM), for all light-OFF sessions pooled together by condition. Note in M1 parkinsonian condition the appearance of small episodes of REM sleep materialized in black on the histogram. *p* < 0.05 * different from healthy OFF-stimulation; # different from parkinsonian OFF-stimulation: Kruskal–Wallis test followed by Dunn’s multiple comparisons test. **e** Typical 30-s example of EEG and EMG tracings showing cortical desynchronization associated with high muscle tone observed in PPN-LFS condition and, **f** cortical synchronization associated with low muscle tone observed in PPN-HFS condition.

A modified multiple sleep latency test **(**mMSLT) was used to measure EDS (Fig. 1f). In all light-OFF sessions, both NHPs fell asleep significantly faster in the parkinsonian state than in the healthy state (M1: 9.7 ± 0.8 min vs. 13.5 ± 0.9 min, *p* < 0.001 and M2: 6.6 ± 1.2 min vs. 11.6 ± 0.8 min*, p* < 0.001 for M2) (Fig. 2a, b). In both NHPs, PPN-LFS restored sleep latency to that observed prior to MPTP treatment. PPN-LFS also induced an increase in sleep latency in the healthy state for M1 (18.5 ± 0.5 min, *p* < 0.001). In the parkinsonian state, EDS was also illustrated by the increased incidence of sleep episodes compared to the healthy state. Indeed, the occurrence of at least one episode of sleep during the light-OFF sessions increased from 77% to 83% for M1 and from 80% to 93% for M2; these percentages significantly decreased with PPN-LFS (83% vs. 63%, *p* = 0.0023 for M1 and 93% vs. 81%, *p* = 0.0370 for M2) (Fig. 2c). In the healthy state, all naps were of short duration and exclusively comprised of non-REM N1 and N2. Whereas in the parkinsonian state, M1 exhibited REM sleep episodes in 45% of the light-OFF sessions with an onset latency of 4.4 ± 0.6 min from the first sleep episode, and M2 exhibited a significant increase in sleep duration (Fig. 2d). These two events were reversed by PPN-LFS; M1’s REM sleep episodes disappeared and the sleep duration decreased in both NHPs (M1: *p* < 0.001 and M2: *p* = 0.0063); promoting a cortical desynchronization associated with a high muscle tone typical of arousal state (Fig. 2e). There was no significant effect of PPN-HFS on wake/sleep behavior despite a tendency to recruit more sleep episodes in light-OFF sessions and thus small episodes of cortical synchronization (Fig. 2f). Therefore, we focused on PPN-LFS rather than PPN-HFS to evaluate how chronic stimulation (12 h of daytime stimulation) influences EDS (Fig. 1g).

**Fig. 3:**
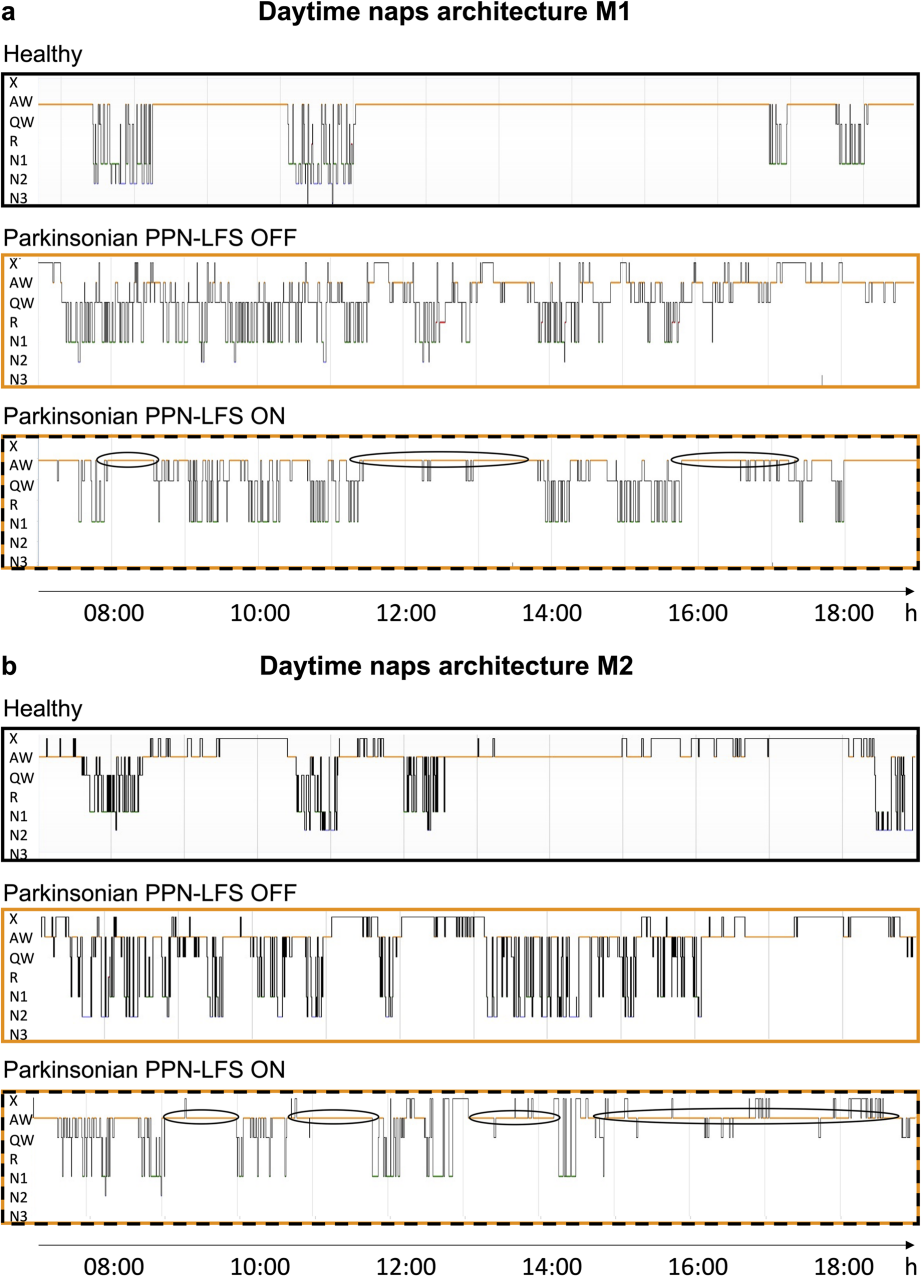
Naps architecture and wake/sleep stages transitions. **a**, **b** Representative daytime hypnogramm for M1 and M2 respectively, during healthy, parkinsonian PPN-LFS OFF and parkinsonian PPN-LFS ON conditions. The line position indicates the sleep stage represented in *y*-axis, with X = artifacts; A = active wake; W = quiet wake; 1,2,3= non-REM sleep stage N1, N2 and N3; R = REM sleep. Note the naps disorganization in parkinsonian state with nearly no continuous active wake periods and the reorganizing effect of PPN-LFS with more active wake periods (circled periods).

**Fig. 4:**
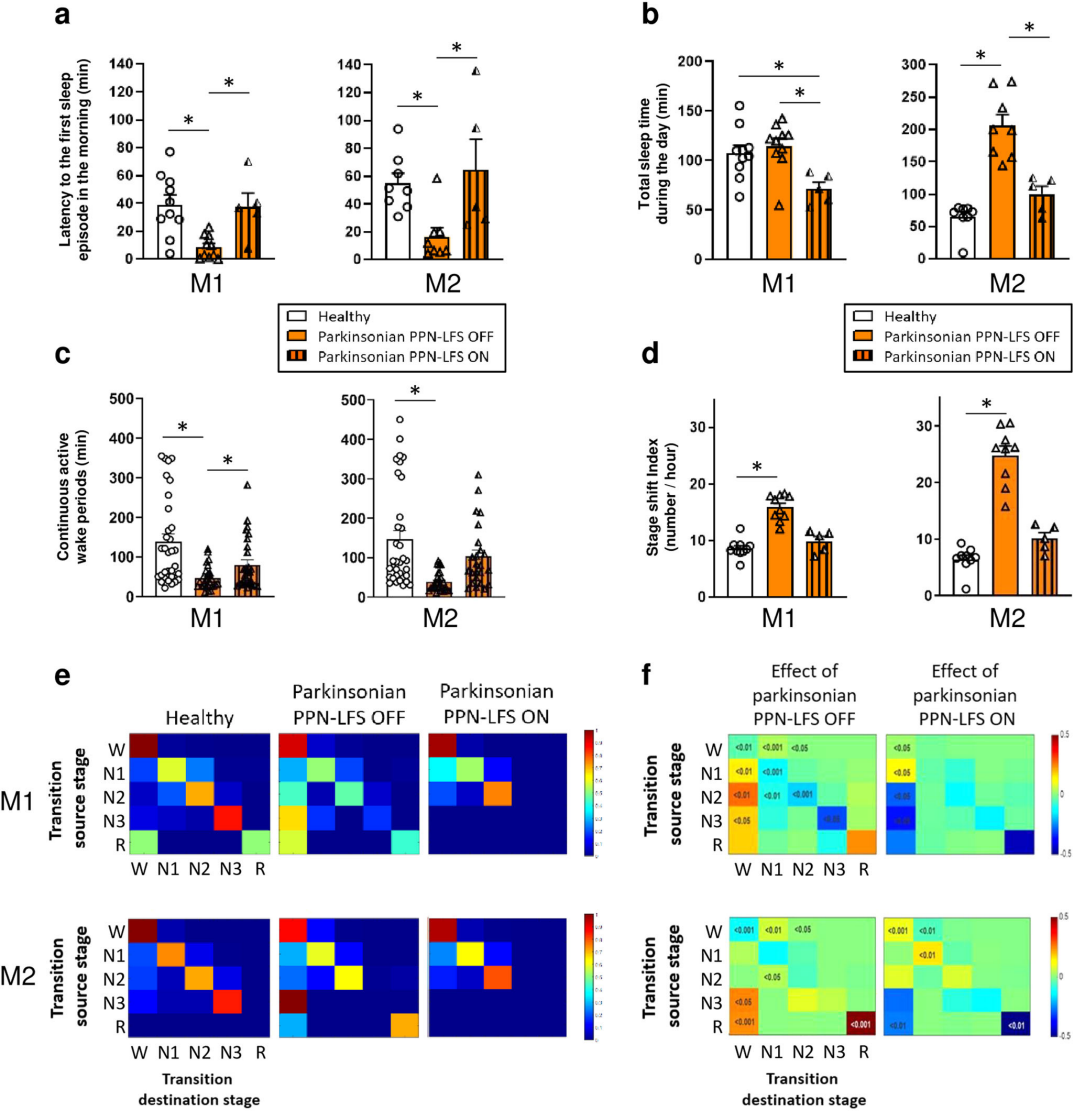
Evaluation of PPN-LFS on excessive daytime sleepiness. These evaluations were performed during healthy (white), parkinsonian PPN-LFS OFF (orange) and parkinsonian with PPN-LFS ON (orange with vertical lines) conditions. **a** Latency to the first sleep episode in the morning for M1 and M2, expressed in minutes (mean ± SEM). **b** Total sleep time during the day for M1 and M2, expressed in minutes (mean ± SEM). **c** Duration of continuous active wake periods for M1 and M2, expressed in minutes (mean ± SEM). **d** Stage shift index for M1 and M2, expressed in number of transition from one stage to another per hour (mean ± SEM). * *p* < *0.05* Kruskal–Wallis test followed by Dunn’s multiple comparisons test. **e** Typical example of matrices of transitions probabilities at healthy, parkinsonian PPN-LFS OFF and parkinsonian PPN-LFS ON. W = wake; 1,2,3= non-REM sleep stage N1, N2 and N3; R = REM sleep. **f** Transitions between wake and sleep stages, source stage (vertical) to destination stage (horizontal) according to the parkinsonian PPN-LFS OFF state (left) and the parkinsonian PPN-LFS ON state (right). Numbers in the matrices are the significant *p* values obtained with Wilcoxon rank sum test.

In healthy animals, daily wake/sleep patterns are characterized by long periods of wakefulness, allowing animals to carry out daily activities, such as food intake, games, or social interaction. The daytime wakefulness of healthy NHPs was interspersed by 3 to 4 naps, usually 2 long morning naps and 1 or 2 short afternoon naps (Fig. 3a, b). In the parkinsonian state, wake/sleep patterns were completely disorganized with naps distributed throughout the day. This disorganization was partially restored by PPN-LFS, with daytime hypnograms showing some restructured naps and an increase in the duration of continuous active wake periods (Fig. 3a, b). The parkinsonian state was also characterized by the appearance a brief occurrence of the first sleep episode in the morning which was significantly restored with PPN-LFS (M1: *p* = 0.0401 and M2: *p* = 0.0317) (Fig. 4a). Despite an increase in small episodes of sleep throughout the day, the total sleep time of M1 was not significantly higher in the parkinsonian state. In contrast, M2 showed a marked increase in total sleep time (Fig. 4b). In both animals in parkinsonian state, a decrease in total sleep time was observed with PPN-LFS compared to the OFF-stimulation condition (M1: 115.0 ± 7.7 min vs. 71.6 ± 8.6 min, *p* = 0.0140; M2: 205.6 ± 17.7 min vs. 100.1 ± 12.5 min, *p* = 0.0121) (Fig. 4b) concomitant with an increase in continuous active wake periods (Fig. 4c) and in the total time spent in active wake (Table 1a).

**Table 1:** Effects of chronic 12 h PPN-LFS on daytime and effects on the following nighttime.

a	DAYTIME					
		M1			M2	
	Healthy	Parkinsonian	Parkinsonian	Healthy	Parkinsonian	Parkinsonian
	(*n* = 10)	PPN LFS-OFF (*n* = 11)	PPN LFS-ON (*n* = 5)	(*n* = 10)	PPN LFS-OFF (*n* = 14)	PPN LFS-ON (*n* = 6)
SL (min)	41.1 ± 6.1	**8.7** ± **2.6** *	**37.9** ± **12.7 #**	55.0 ± 7.2	**16.6** ± **6.4** *	**64.6** ± **21.8 #**
REM latency (min)	69.3 ± 4.6 (30%)	47.3 ± 12.1 (81.8%)	N/A (0%)	582.5 (10%)	161.6 ± 46.4 (42.9%)	N/A (0%)
# SOREMP/day	0 ± 0	1.7 ± 0.4	0 ± 0	0 ± 0	2.0 ± 0.4	0 ± 0
TST (min)	107.4 ± 8.2	115.0 ± 7.7	**71.6** ± 8.6 *#	64.8 ± 8.1	**205.6** ± **17.7** *	**100.1** ± **12.5 #**
% Stage 1	8.6 ± 1.1	**11.0** ± 0.8 *	8.6 ± 1.3	6.0 ± 0.9	**18.4** ± **1.2** *	**9.9** ± **0.9 #**
% Stage 2	5.0 ± 0.4	3.7 ± 0.5	**1.3** ± 1.2 *#	2.9 ± 0.6	**9.1** ± **2.0** *	**4.0** ± **1.6 #**
% Stage 3	1.1 ± 0.3	**0.2** ± **0.1**	0.0 ± 0.0	0.0 ± 0.0	0.1 ± 0.05	0.0 ± 0.0
% REM sleep	0.2 ± 0.1	**1.2** ± **0.3** *	0.0 ± 0.0	0.1 ± 0.1	**1.0** ± **0.2** *	0.0 ± 0.0
% AW	80.6 ± 1.4	**50.5** ± **2.4** *	**65.3** ± **1.6 #**	85.8 ± 1.8	**61.6** ± **2.0** *	**75.8** ± **1.8 #**
% QW	4.4 ± 0.6	**27.3** ± **1.7** *	24.8 ± 1.8 *	5.3 ± 0.8	**9.7** ± **1.1** *	10.2 ± 0.6 *

b	NIGHTTIME					
		M1			M2	
	Healthy	Parkinsonian	Parkinsonian	Healthy	Parkinsonian	Parkinsonian
	(*n* = 10)	(*n* = 15)	Post PPN LFS-ON (*n* = 5)	(*n* = 10)	(*n* = 14)	Post PPN LFS-ON (*n* = 6)
SL (min)	13.4 ± 1.5	18.7 ± 2.8	34.9 ± 5.8 *	7.5 ± 1.2	4.8 ± 1.5	7.1 ± 1.3
REM latency (min)	70.3 ± 12.9 (100%)	23.9 ± 8.9 (100%)	**184.9** ± **31.7 #** (100%)	49.2 ± 11.7 (100%)	31.5 ± 12.9 (100%)	60.9 ± 24.9 (100%)
TST (min)	523.1 ± 5.1	**230.4** ± 7.0 *	281.9 ± 17.2 *	589.4 ± 9.3	**419.9** ± 14.0 *	438.6 ± 5.7 *
% Stage 1	18.2 ± 1.0	**13.3** ± 1.1 *	**16.7** ± **0.9 #**	20.0 ± 2.0	**31.5** ± 0.9 *	33.7 ± 1.4 *
% Stage 2	17.0 ± 2.0	**8.2** ± 0.4 *	12.6 ± 1.2 *	32.1 ± 2.2	**14.6** ± 2.0 *	14.4 ± 0.7 *
% Stage 3	25.2 ± 1.5	**5.4** ± 0.6 *	4.8 ± 0.9 *	18.2 ± 1.7	**5.1** ± 0.9 *	6.5 ± 0.9 *
% REM sleep	12.4 ± 0.4	**5.1** ± 0.4 *	5.1 ± 0.6 *	11.4 ± 0.7	**7.1** ± 0.7 *	5.6 ± 0.4 *
% AW	17.2 ± 0.8	**38.0** ± 1.3 *	33.43 ± 1.2 *	15.5 ± 1	**28.0** ± 2.1 *	26.8 ± 0.5 *
% QW	10.0 ± 0.8	**29.0** ± 1.2 *	26.2 ± 1.3 *	2.7 ± 0.5	**13.6** ± 2.3 *	13.4 ± 1.1 *
WASO (min)	182.3 ± 5.2	**465.8** ± 6.7 *	394.6 ± 10.6 *	120.1 ± 9.3	**289.4** ± 13.3 *	271.9 ± 7.3 *
Sleep efficiency (%)	72.7 ± 0.7	**32.0** ± 1.0 *	39.2 ± 2.4 *	81.9 ± 1.3	**58.3** ± 1.9 *	60.9 ± 0.8 *
Sleep cycle number	11.4 ± 0.3	**0.8** ± 0.4 *	2.4 ± 0.8 *	11.4 ± 0.6	**5.7** ± 0.7 *	6.4 ± 0.7 *

Each column represents mean values (± SEM) derived from 12 h long-term recordings during **a)** the daytime in healthy, parkinsonian and parkinsonian PPN-LFS ON conditions and **b)** the nighttime in healthy, parkinsonian and parkinsonian post PPN-LFS conditions. For daytime, SL refers to the latency to the first sleep episode in the morning and for nighttime SL corresponds to the time in minute between the light turned off (19:00 h) and the first sleep episode. REM latency corresponds to the time in minute between the first sleep episode and the first REM sleep episode; numbers in parenthesis indicate the percentage of recordings including at least one REM sleep episode allowing to calculate the latency. For daytime, the number of sleep onset REM period (# SOREMP/day) refers to the number of times per day that REM sleep episode occurred within 15 min of sleep. TST refers to the total sleep time during the 12 h recording expressed in minute. The amounts of each stage of wake and sleep are expressed as the mean percentage of the total scoring time. WASO, expressed in minute, refers to the sleep period time minus the TST. Sleep efficiency, expressed in percentage, was defined as the ratio of TST to the 12 h nocturnal time. *p* < 0.05 *different from healthy state, # different from parkinsonian state: Kruskal–Wallis test followed by Dunn’s multiple comparisons test. Bold numbers indicate significant differences to be highlighted between two similar conditions with only one changing parameter.

*SL* sleep latency, REM rapid-eyes movement, *TST* total sleep time, *AW* active wake; QW quiet wake.

Daytime naps, mainly composed of non-REM sleep N1 and N2 in healthy state, were additionally composed of small episodes of REM sleep in the parkinsonian state; these REM episodes were abolished with PPN-LFS (Table 1a). Although the overall REM sleep latency was not significantly altered in the parkinsonian state, the number of sleep onset REM periods per day (#SOREMP/day) was increased and returned to healthy value with PPN-LFS (Table 1a). Moreover, PPN-LFS induced a decrease in time spent in N2 for both animals (M1: 3.7 ± 0.5% vs. 1.3 ± 1.2%, *p* = 0.0307; M2: 9.1 ± 2.0% vs. 4.0 ± 1.6%, *p* = 0.0265), associated with a decrease in spindle density per 30 seconds of N2 sleep stage without affecting their duration (Supplementary Fig. 2a–c).

The diurnal sleep disorganization in parkinsonian animals was also highlighted by a significant increase in the stage shift index (Fig. 4d) and a significant increase in wake/sleep transitions (Fig. 4e, f). We found a significant increase in the number of transitions from wakefulness to non-REM sleep N1 and N2; conversely, there was an increase in transitions from sleep states to wakefulness (Fig. 4e, f). This daytime wake/sleep disruption was partially reversed with PPN-LFS. Thus, the decrease in transitions from sleep states to wakefulness with PPN-LFS demonstrates a reorganization of diurnal sleep architecture, and the significant increase in the transitions from wake state to itself with PPN-LFS highlights an increase in the time spent awake without sleep episode interruption (Fig. 4e, f).

### Nocturnal sleep quality

In the parkinsonian state, animals showed significant alteration of nighttime sleep with a significant decrease in total sleep time and an increase in wake time after sleep onset, which led to a significant decrease in sleep efficiency (Table 1b). These animals showed a strongly disorganized sleep architecture with a decrease in N2, N3 and REM sleep. Nights after a full day of PPN-LFS show an increase in sleep latency in M1 and did not exhibit significant changes in sleep quality. However, for both NHPs there was a tendency toward an increase in total sleep time, especially for non-REM sleep N1 which is significant for M1, and a decrease in the wake time after sleep onset (Table 1b).

## Discussion

The NHPs exhibited significant altered wake/sleep behavior in the parkinsonian state compared to the healthy state (as already described in different NHP model of PD^30,31,33^ and also observed in patients with advanced PD^34,35^). MPTP-treated animals showed EDS, characterized by a decrease in sleep latency, an inability to maintain long periods of wakefulness, and prominent disorganization of naps. During the nighttime, sleep quality was altered, with a dramatic decrease in total sleep time and in sleep efficiency. In addition to further confirm the emergence of wake/sleep behavior disturbances in the MPTP-treated monkey model, this study demonstrates that in this model, it is possible to modulate wake/sleep behavior using DBS therapy. Indeed, this study shows strong evidence that PPN-LFS significantly reduces EDS.

The mMSLT measures the tendency of an individual to fall asleep during the daytime under controlled conditions. This test is based on the notion that sleep latency reflects underlying sleepiness^36^. Overall, in parkinsonian OFF-stimulation condition, sleep latency decreased in both animals which is a marker of daytime sleepiness^37^; and PPN-LFS restored sleep latency prior to MPTP treatment and even decreased sleep duration during light-OFF sessions of mMSLT. The improvement in EDS with PPN-LFS was more pronounced in M1 who also exhibited this effect in the healthy state. This difference between the animals can be explained by the use of a higher voltage in M1 than in M2 and thus a higher surface charge density. It is difficult to make modeling effect of the stimulation with the type of lead used; however, we can hypothetize that using this experimental lead, and those experimental stimulation settings at a mean impedance of 1898 Ω for M1 and 1645 Ω for M2 it is very likely that the electrode stimulated in a very close vicinity around the contact, mainly the PPN area, but perhaps neighboring structures also involved in arousal such as the nucleus pontis oralis^38^ and the cuneiformis nucleus^39^ in M1 at a higher voltage. In contrast, PPN-HFS did not produce any significant effect; we observed only a slight tendency toward an increase in the incidence of sleep episodes, especially in the healthy state, which is consistent with experimental studies in rats^40^. The reticular configuration of the PPN may explain this discrepancy between the effects of HFS and LFS. We assumed that HFS would induce sleep by inhibiting the PPN, which is an arousal-related structure. However, arousal involves more than one brain structure, which may explain why sleep induction and maintenance are difficult to achieve through neuromodulation of the PPN alone. Thus, PPN-DBS should be considered a more robust therapeutic option to induce wakefulness rather than to restore sleep in humans.

In the morning, animals fell back asleep significantly faster in the parkinsonian state and, although not significant, a decrease in REM sleep latency was also observed. We speculate that the non-significance results from the low sample values of REM sleep latency in healthy condition given that REM sleep occurred in only 30% and 10%, for M1 and M2 respectively, of the long-term 12 h recordings. However, in the parkinsonian state the occurrence of REM sleep was increased, and SOREMP were observed in both animals. SOREMP, defined as REM sleep periods that occur within 15 min of sleep onset, are a specific marker of narcolepsy^41^, a disease that shares similarities with wakefulness disorders and notably EDS. In PD, it has been proposed that EDS can occur as an isolated symptom^42–44^, but is also associated with the various forms of nocturnal sleep disorders. Indeed, PD patients frequently experience different phenotypes of nocturnal sleep disorders, such as insomnia^45^, REM sleep disorders^46^ or sleep apnea syndrome^47^. In our model, the observed poor quality of nighttime sleep by itself may explain the observed EDS. However, a recent study showed that improved nighttime sleep in PD patients does not affect daytime sleepiness, suggesting that EDS may occur independently of nighttime sleep disturbances in PD patients^44^. EDS, which has a significant impact on quality of life, merits better management. In the present study, we showed that PPN-LFS applied throughout the day (12 h PPN-LFS) restored a more typical diurnal wake/sleep pattern and decreased the total sleep time, in both parkinsonian animals. Indeed, this therapy induced a greater and more physiologically typical range of arousal. The duration of sleep returned to physiological values in M2 and was even decreased in M1 compared to the healthy state after chronic PPN-LFS. Despite a strong effect on sleep latency and duration of sleep during the day, the moderate effect on naps organization observed on hypnograms suggests that the PPN plays only a partial role within the different arousal systems. It could be argued that the increase in wakefulness with PPN-LFS works through the functional activation of ascending neurons in the PPN which, in turn, activated the thalamus and its widespread projections to the cortex^48,49^ that facilitated cortical desynchronization, which is typical during wake state. Our preliminary study on the cortical spindles activity showed that PPN-LFS decreased the spindles density per 30 s during non-REM sleep N2, without affecting their duration (Supplementary Fig. 2a–c), which demonstrates that the thalamo-cortical network is partly involved in the arousal promoting effect of PPN-LFS. This analysis deserves to be extensively investigated in future studies.

The close link between wakefulness and sleep led us to believe that increases in daytime wakefulness would have repercussion on sleep quality; however, we did not observe any significant improvement in nighttime sleep quality after chronic daytime PPN-LFS, despite a tendency in that direction. This observation is consistent with a study carried out in humans showing that improving the nighttime sleep quality with medication does not change the quality of wakefulness, i.e., EDS is still present^44^. Nevertheless, due to the strong interaction between the mechanisms underlying wakefulness and sleep, one might expect that the daytime application of long-term chronic PPN-LFS could contribute to improving sleep quality. This hypothesis can be tested in future studies.

In PD patients, neuronal degeneration largely targets the dopaminergic system, and is further extended to other brain regions, such as the cholinergic neurons in the PPN^50,51^. This major observation raises the question of the effectiveness and utility of stimulating an area in which the neurons have degenerated. However, a recent study showed that the specific activation of cholinergic neurons by designer receptors exclusively activated by designer drugs (DREADDs) reversed motor deficits in a rat model of PD that mimicked the degeneration of dopaminergic neurons in the substantia nigra and cholinergic neurons in the PPN^52^. This finding could suggest that chronic stimulation of the remaining cholinergic neurons and also other neuronal populations could effectively reduce disturbances in wake/sleep patterns.

Histological analyses showed that the stimulating contacts of the electrodes were located in the lateral edge of the PPN area, apart from the medial lemniscus, in both animals. However, the closer proximity of the electrode from this structure in M2 could explain why the threshold for side effects was lower for this animal, and therefore why we could not increase the voltage as was done for M1. In humans, the proximity of the PPN to the medial lemniscus often results in sensory symptoms such as paresthesia, which are the most common side effects of PPN-DBS^53^. One could argue that the effect observed on EDS could be explained by the electrical stimulation diffusion to the medial lemniscus that could maintain animals awake due to unpleasant side effect. If this hypothesis was true, we should have observed the same side effect at low and high frequencies. However, the awakening effect was observed only at LFS; not at HFS. It is difficult to assess and quantify paresthesia in NHPs. However, we proceeded with careful observation and did not observe any sign of animal discomfort during stimulation tests. PPN-HFS at high intensity induced myoclonus in M1, as previously reported in humans^54^, and contralateral hemifacial contraction in M2, which could eventually indicate paresthesia as previously reported in humans as well^54^. In any case, stimulations were performed at a voltage set 20% below the threshold for side effects and HFS (condition in which the density of the delivered current is the highest) did not generate, at any moment, signs of discomfort. Animals were even quieter and able to sleep in this condition. For these reasons, it is very unlikely that the awakening effect observed at LFS is due to a side effect related to current diffusion to the medial lemniscus. These observations support a specific effect of PPN-LFS on the control of arousal behavior.

We acknowledge the limitations of translating experimental animal data to human conditions. The use of animal models cannot perfectly mimic the human disease. However, the MPTP-treated NHPs model exhibited sleep/wake disturbances similar to that observed in PD patients. Thus, MPTP-treated NHPs experience disorganization of nighttime sleep with reduced sleep quality and EDS characterized by sleep episodes occurring more rapidly in the morning and spreading through the middle of the day, as described in a recent study conducted by our group^33^ and others^30,31,55^. We also acknowledge the limited number of animals used in the present study, due to ethical considerations concerning animal use; however, our results were consistent across animals.

The present study confirmed that it is possible to modulate wakefulness in an NHP model of PD using PPN-LFS and confirmed previous observations in humans^29^. The arousal-inducing effect of PPN-LFS could contribute to the development of new therapeutic approaches for treating severe EDS that significantly impairs the quality of life of patients with neurodegenerative diseases such as PD and Alzheimer’s disease as well as patients with central hypersomnolence disorders such as narcolepsy.

## Methods

### Animals

In accordance with the policy of Grenoble Alpes University and the Grenoble Institut of Neurosciences (B3851610008) and with French legislation, experiments were performed in compliance with the European Community Council Directive of 2010 (2010/63/UE) for care of laboratory animals. All procedures were reviewed and validated by the “Comité éthique du GIN n˚004” and was authorized by the Direction Départementale des Services Vétérinaires de l’Isère—Ministère de l’Agriculture et de la Pêche, France. The study was performed on two adult male NHPs (*Macaca fascicularis*—CRP Port Louis, Mauritius*)*, 8–10 kg and both 10 years of age. Animals were kept under controlled conditions, 12-h light/dark cycles [light off at 19:00 h], 23 ± 2 °C, and 50 ± 5% humidity. Animals were pair housed with other NHP, had access *ad libitum* to food and water and supplemental fresh fruit and vegetable was given once a day.

### Apparatus

NHPs were chronically implanted with a polysomnographic equipment, a radio-telemeter transmitter (D70-EEE, Data Science International, France) for continuous and long-term recording in freely moving animals. The transmitter had three channels biopotential device for recording electro-encephalogram (EEG), electro-oculogram (EOG), and electro-myogram (EMG) signals with a sampling rate of 500 Hz and a gain of 75. Polysomnographic biopotential signals were acquired via two receivers mounted in the home cage and behavioral cage and then forwarded to a data exchange matrix connected to a computer for data recording and storage (Dataquest A.R.T., Data Sciences International, France). The transmitter also records actimetry data, representing the ambulatory locomotor activity of animals. NHPs were videotaped concurrently with the recording of polysomnographic and actimetry signals; allowing to validate the sleep scoring and to make motor evaluation in offline analysis. The animals were also chronically implanted with a deep brain electrode in the PPN area. A quadrupole electrode lead (length 20 mm, contact length 0.5 mm, spaced 0.5 mm apart, outer diameter 0.8 mm, Dixi, Besancon, France) was stereotactically implanted into the PPN area and connected to a neurostimulator (Activa®PC + S, Medtronic, Minneapolis, USA) thanks to a lead extension (37086, 40 cm, Medtronic, Minneapolis, USA). The stimulator could be turn on and off using a pulse generator programmer (Medtronic, 8840 Programmer) by placing the transmitter against the implanted stimulator. Animals were trained to this manipulation in their home cage, without any sedation.

### Surgery

The surgery was performed under aseptic conditions and general anesthesia. Animals were initially anesthetized with Ketamine (7 mg/kg, i.m.) and Xylazine (0.6 mg/kg, i.m.) then intubated and switched to isoflurane mixed with 100% oxygen. The animals were spontaneously breathing and placed in the stereotaxic frame (Kopf, CA). Respiration rate, Et-CO_2_ and O_2_ saturation were monitored with a Comdek MD-660P monitor. Saline solution (NaCl 0.9%, Sigma, France) was infused intravenously all along the duration of the surgery for drug access and hydration. The radio-telemeter transmitter was implanted within the abdominal muscle layers and the electrodes biopotential leads were tunneled subcutaneously to the skull. EEG was recorded using two electrodes screwed unilaterally (one frontal and one parietal 10 mm lateral to the midline at right) into the skull, EOG was acquired from two electrodes affixed at the level of the right orbital arch bone unilaterally (one at the top and one at the external side) and finally EMG was monitored from two leads sutured into the right neck musculature at 10 mm apart. The reference was fixed on the skull at the left occipital level. The neurostimulator was implanted in a subcutaneous pocket in the back, the lead extension was tunneled subcutaneously to the skull and the quadrupole electrode was implanted into the right PPN area. For this purpose, a preoperative MRI (3D T1 sense, performed at Grenoble MRI facility IRMaGE) resurfaced in the stereotaxic atlas of the macaque fascicularis^56^ allowed us to define the coordinates of the target structure relative to internal anatomical landmarks: the reference line connecting the anterior commissure and the posterior commissure (AC-PC line). The target was determined at −12mm posterior to AC, at 3 mm lateral to the midline and 6 mm below the AC-PC line. Then, based on a ventriculographical X-ray control, using a cannula placed on the left lateral ventricular through which 2 ml of ventricular contrast (Iopamiron 200, iodine 200 mg/ml, Bracc) was injected, the electrode was anchored in situ with acrylic dental and suture and then connected to the stimulator. Analgesic/anti-inflammatory therapies (Ketoprofen 2 mg/kg i.m.) and antibiotic cover (Clamoxyl, 20 mg/kg i.m.) were provided during the one-week post-operative period.

### PPN stimulation setting

Continuous PPN-DBS was applied unilaterally with parameters selected from a stimulation range carried out on awake animals, from 5 Hz to 130 Hz for each contact of the electrode. These parameters were chosen based on the most relevant behavioral effects (such as a change in muscle tone, acute activity, state of consciousness or the presence of yawning) and were similar to those routinely used in clinic (LFS at 20 Hz and HFS at 80 Hz, 60 µs of pulse width). Voltage was determined on the basis of the voltage threshold that induced side effect for each animal and each contact (contralateral myoclonus for M1 and contralateral hemiface contraction and nausea for M2). For both animals, the two middle contacts were considered as the optimal contact within the PPN area and the most suitable for chronic stimulation. The stimulation was delivered in a bipolar mode at voltage set at 80% of the side effect threshold value (LFS and HFS: 5.6 V for M1, LFS: 3 V and HFS: 1.8 V for M2). In these settings, no abnormal behavior indicating discomfort was observed.

### Estimation of charge densities

The charge densities at the electrode surface were estimated by dividing the total current by the surface area of the 2 bipolar contacts used (1.2mm^2^). The charge densities were 0.15 µC/mm^2^ for M1 and 0.09 µC/mm^2^ for M2, calculated according to Eq. (1). The impedances of the contacts used were taken every 2 months throughout the experiments, we could observe a stability with average impedances of 1898Ω for M1 and 1645Ω for M2.

1 $$\frac{{\left[ {\frac{{{\rm{Voltage}}\left( V \right)}}{{{\it{{\rm{Resistance}}}}\left( {\Omega} \right)}}} \right]\ast {\rm{Pulse}}\,{\rm{Width}}\left( {\mu {\rm{s}}} \right)}}{{{\rm{Area}}\left( {{\rm{mm}}^2} \right)}} = {{{\rm{Charge}}}}\,{\rm{Density}}\left( {\frac{{\mu {\rm{C}}}}{{{\rm{mm}}^2}}} \right)$$

### MPTP treatment and motor score evaluation

After collecting the baseline data, monkeys were treated by intramuscular injection of MPTP under light anesthesia (Ketamine 2–4 mg/kg). We used an original protocol consisting in a progressive administration of small doses of MPTP (0.2–0.5 mg/kg, in NaCl 0.9%) at an interval of 2 weeks until the emergence of a stable parkinsonian syndrome. Monkey 1 received 18 injections (7.55 mg/kg total) and monkey 2 received 8 injections (2.2 mg/kg total) to achieve comparable parkinsonian syndrome.The severity of parkinsonian syndrome was evaluated before, during and after MPTP treatment in the home cage, using a rating scale, combining the most recurring items from eight commonly used parkinsonism scales^57^. This scale includes 8 clinical symptoms (general activity, frequency of each arms movements, posture, bradykinesia, tremor, feeding, freezing and vocalization), rated between 0 (normal) and 2–3 (depending on the degree of disability), with a total score out of 25 (maximal parkinsonian severity). Evaluations were performed by the same observer twice a week at 14:00 h for 15 min.

### Sleep data analysis

Sleep scoring was blindly performed offline on a dedicated software (NeuroScore, Data Science international, France). The scoring of sleep stages was manually performed according to the American Academy of Sleep Medicine criteria and was performed, as in humans, in 30 s epochs, so each one were assigned to a single stage. EEG and EOG was bandpass-filtered in the range of 0.3 to 35 Hz and EMG was bandpass-filtered in the range of 10 to 100 Hz. The different stages identified were “active wake”, “quiet wake” (with alpha waves), animal calm and usually has closed eyes, “non-REM sleep” stage 1 (with theta waves and vertex sharp waves) and stage 2 (with theta waves and K complex/spindles), “deep sleep” stage 3 (with delta waves) and REM sleep (Fig. 1h). Movement and chewing artifacts were mostly produced during wakefulness and all epochs containing them were considered as “active wake” after video reviewing.

### Excessive daytime sleepiness evaluation: mMSLT

Daytime sleepiness was evaluated using mMSLT, as used in clinic but adapted to monkeys, performed 2 h after waking up (Fig. 1f). In a quiet room, for each mMSLT, lights were turned off 3 times (light-OFF sessions at 10:00 h, 11:00 h, and 12:00 h) for a duration of 20 min. Between light-OFF sessions, lights were kept ON and every effort was made to keep monkeys awake. In healthy state, we randomly tested the effect of OFF-stimulation (level of normal daytime sleepiness), PPN-LFS and PPN-HFS respectively 10, 5 and 5 times for each animal (resulting in 30 values for healthy OFF-stimulation condition and 15 values for each healthy ON-stimulation conditions). Next, mMSLT were performed once stable parkinsonian syndrome was established and then, coupled to the same stimulation parameters at LFS and HFS, repeated minimum 10 times for each condition which corresponds to 30 values for OFF-stimulation and ON-stimulation conditions. Sleep latency was determined if a 30 s epoch of scorable sleep was observed. If no sleep onset was observed, sleep latency was designated to be 20 min, and the lights were turned back ON until the next light-OFF session. The two wake stages identified: “active wake” and “quiet wake” were pooled together. Sleep was scored in light sleep (stages 1 and 2 of non-REM sleep pooled together). The following parameters were calculated for each 20 min light-OFF session: mMSLT sleep latency (min) for distinct light-OFF session, mean sleep latency (min) by averaging all light-OFF sessions, incidence of at least one 30 s sleep episode (%) and sleep duration (min). Data were processed individually for M1 and M2.

### Excessive daytime sleepiness evaluation: long-term recordings

Assessment of daytime sleepiness was done by recording of 12 h from 7:00 h to 19:00 h (Fig. 1g). These recordings were made on weekends to be sure to obtain spontaneous behavior, limiting external factors such as noise and the passage of users. We collected a total of minimum 10 recordings in the healthy and parkinsonian state and 5 recordings for PPN-LFS (ON-stimulation from 7:00 h to 19:00 h). For each 12 h period, the latency to the first sleep episode in the morning (min), the REM sleep latency (min), the number of sleep onset REM sleep periods per day (#SOREMP/day) indicating the number of times per day that REM sleep episode occurred within 15 min of sleep, the total sleep time (min), the relative duration of each stage (%) and the duration of continuous active wake periods (min) were calculated. Data were processed individually for M1 and M2.

#### Wake/sleep stages transitions analysis

The stage shift index, defined as the number of transitions between sleep stages per hour, were calculated for all 12 h recording sessions from 7:00 h to 19:00 h and for all conditions. This parameter was used as an index of overall arousal fragmentation in this study. The significance was test with a Kruskal–Wallis test followed by Dunn’s multiple comparisons test. We used a Markov analysis method to study the transitions between the wake/sleep stages during the day. This analysis assumes that the probability of change in stage within the next epoch is dependent only on the stage in the current epoch (first-order dependence) and is neither independent (zeroth-order dependence) nor dependent on the stage of previous epochs. We used five stages (wake, N1, N2, N3 and REM) to build these transition maps of probability to go from one stage to the other^58^. Thus, the probability for each row had a sum of 1 if the sleep stage was reach at least once during the experiment and was set at 0 otherwise.

### Nighttime sleep quality post-PPN-LFS evaluation

Nighttime sleep data were recorded from 19:00 h to 7:00 h the night following the daytime recordings. We recorded 10 nights during the healthy and parkinsonian states and 5 nights following the daytime PPN-LFS. For each night, the sleep latency (min), the REM sleep latency (min), the total sleep time (min), the relative duration of each stage (%), the wake time after sleep onset (min), the sleep efficiency (expressed in %, as the ratio of the total sleep time to the 12 h nocturnal time) and the number of sleep cycle were calculated.

### Immunohistochemistry

Animals were deeply anesthetized with ketamine and pentobarbital (10 mg/kg and 25 mg/kg i.m.), then transcardially perfused with 0.9% saline solution followed by 4% paraformaldehyde in 0.1 M phosphate buffer (PB), pH 7.4. Brains were removed from the skulls, post-fixed for 24 h and cryoprotected in sucrose density gradient (from 10% to 30%). 50 µm free-floating sections were cut, using a cryotome, and store at -20 °C in a cryoprotective solution until immunochemistry.

To determine the location of the electrodes, Cresyl violet staining was performed on sections where the electrode track was visible. Brain sections, 50 µm thick, were mounted on gelatin-coated slides, degreased with xylene and rehydrated in decreasing ethanol baths (100%, 95%, and 70%). Sections were then stained with a 1% Cresyl violet solution for 4 min and then dehydrated in increasing ethanol baths (70%, 95% and 100%) before being placed in a xylene degreasing solution for 30 min. Sections were then coverslipped and image acquisition was done with a slide scanner (Manufacturer: Carl Zeiss; Model: Axioscan Z1; Software: ZenZEISS). Then, the deeper visible trace allowed us to position the proximal tip of the electrode and reposition the contacts with the electrode measurements described in the apparatus section.

To assess the dopamine depletion, regularly spaced sections, with the substantia nigra and striatum including the caudate nucleus and putamen, were taken at comparable levels from each brain and compared with a control brain from the tissue bank available at our institute. After washing they were incubated 15 min with 10% methanol and 3% hydrogen peroxide in 0.1 M PB to inhibit endogenous peroxidase activity. After several rinses, they were placed at least 2 h at room temperature, in blocking solution containing 10% normal goat serum in 0.1 M PB with 0.3% triton X100. Then the sections were incubated at 4 °C for 48 h with rabbit anti-TH (AB152, Sigma-Merck, France; 1:1000 in 5% normal goat serum in 0.1 M PB triton 0.3%) followed by biotinylated goat anti-rabbit IgG (1:500 in 0.1 M PB triton 0.3%) for 1h30. After washing, the sections were incubated for 1 h with an avidin-biotin peroxidase complex (ABC standard kit; Vector Laboratories). The bound peroxidase was visualized with diaminobenzidine solution as chromogen (SK-4100, Vector Laboratories). TH-immunolabelling detection of dopaminergic neurons and terminals were evaluated under a slide scanner (Manufacturer: Carl Zeiss; Model: Axioscan Z1; Software: ZenZEISS) and then analyzed with the ICS FrameWork computerized image analysis system (TRIBVN, 2.9.2 version, Châtillon, France). For quantification, the selected TH-labeled coronal sections for each experimental animal correspond to the AC + 2 mm level for the striatum and to the AC-6mm level for the substantia nigra, according to the stereotaxic atlas^56^. Optical densities were measured for each striatal and substantia nigra subregion, and the mean optical densities was calculated with ICS FrameWork software (TRIBVN, 2.9.2 version). Optical densities values were measured for the denervated and non-denervated territories of the MPTP-treated animals for each section analyzed and were compared with those for the homologous regions in control animal. The optical densities value obtained for an unlabeled area (ventricle or lenticular fasciculus) was used as the background and was subtracted from each of the optical densities values measured.

### Statistical analysis

Standard statistical methods using GraphPad Prism 9 software were applied. A Kruskal–Wallis test followed by Dunn’s multiple comparisons test was used for the comparison of sleep parameters in the healthy state and after MPTP treatment in the parkinsonian state and then in the different stimulation conditions: PPN-LFS and -HFS. A Fisher’s exact test was performed to determine if the incidence of sleep episode was depending on the PPN-DBS conditions. The difference between transition matrix obtained in the healthy state, in the parkinsonian state and parkinsonian state with PPN-LFS were evaluated using a Wilcoxon ranks sum test. Data are presented as mean ± standard error of the mean (SEM) and the statistical significance was considered at a probability (p) value ≤ 0.05.

### Reporting summary

Further information on research design is available in the Nature Research Reporting Summary linked to this article.

## Supplementary information

Supplemental information

Reporting Summary

## Data Availability

All data reported in this article can be shared on reasonable request from qualified investigators by contacting the corresponding author.
