# Supplementary material for: Excessive daytime sleepiness in a model of Parkinson’s disease improved by low-frequency stimulation of the pedunculopontine nucleus
Source: NPJ Parkinsons Dis. 2023 Jan 25;9:9. doi: 10.1038/s41531-023-00455-7 (PMC9876933; doi:10.1038/s41531-023-00455-7)
Supplement: Supplementary file 1 — Supplemental information [file 41531_2023_455_MOESM1_ESM.pdf]

Supplementary information

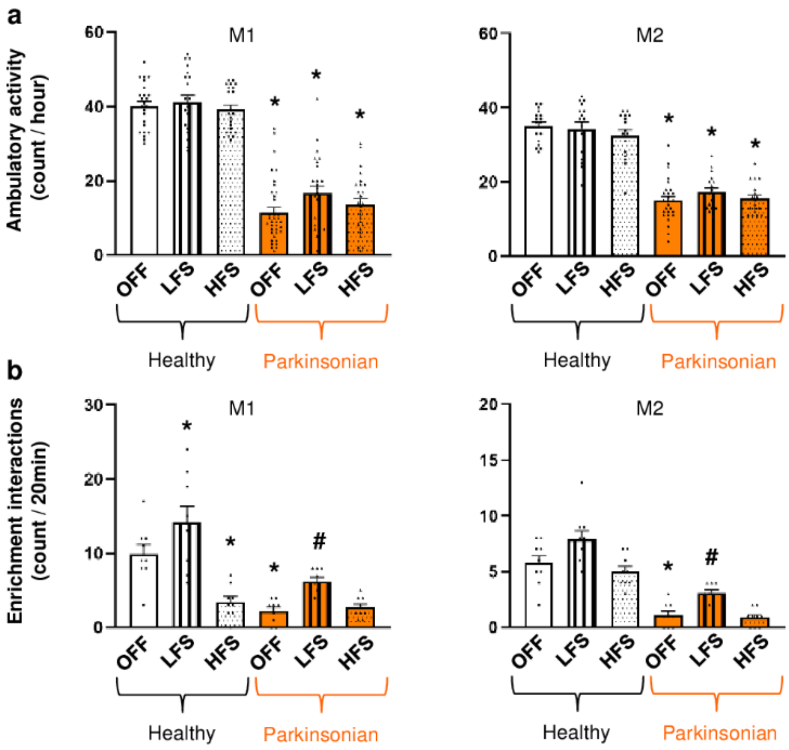

Supplementary Figure 1: Effect of PPN-LFS and -HFS on motor behavior in parkinsonian animals. a Ambulatory locomotor activity, expressed in count per hour (mean ± SEM), calculated at fixed time (10:00 h) in healthy (white) and parkinsonian (orange) states, with PPN-LFS (vertical lines) or PPN-HFS (dots), repeated minimum 18 times for M1 (left) and M2 (right). **b** Number of interactions with the enrichment (mean ± SEM) present in the behavioral cage during 9 random light-OFF sessions for M1 (left) and M2 (right). *different from healthy state OFF-stimulation, # different from parkinsonian state OFF-stimulation: Kruskal-Wallis test followed by Dunn's multiple comparisons test was used to determine whether motor changes were dependent on the different PPN-DBS conditions.

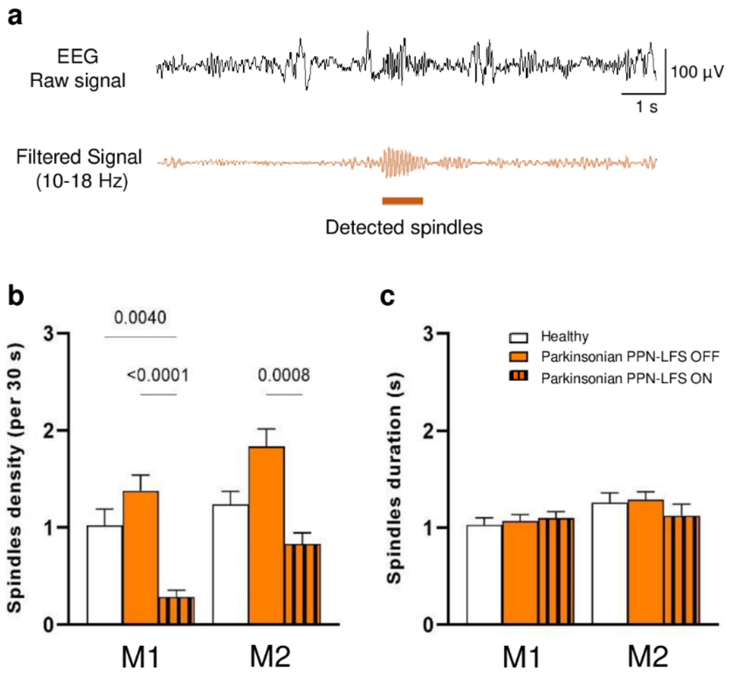

Supplementary Figure 2: Basic features of sleep spindles during non-REM sleep stage 2. a Example of a recorded spindle events. **b** Spindles density per 30 s (mean ± SEM) during non-REM sleep N2 for M1 and M2 in healthy (white), parkinsonian (orange) and parkinsonian with PPN-LFS (orange with vertical lines) conditions. **c S**pindles duration**,** expressed in second (mean ± SEM). A Kruskal-Wallis test followed by Dunn's multiple comparisons test was used to compare in the healthy, parkinsonian and parkinsonian with PPN-LFS conditions.
