# Supplementary material for: Excessive daytime sleepiness in a model of Parkinson’s disease improved by low-frequency stimulation of the pedunculopontine nucleus
Source: NPJ Parkinsons Dis. 2023 Jan 25;9:9. doi: 10.1038/s41531-023-00455-7 (PMC9876933; doi:10.1038/s41531-023-00455-7)
Supplement: Supplementary file 2 — Reporting Summary [file 41531_2023_455_MOESM2_ESM.pdf]

Corresponding author(s): Brigitte Piallat
brigitte.piallat@univ-grenoble-alpes.fr

Last updated by author(s): 2022/05/18

Reporting Summary

Nature Portfolio wishes to improve the reproducibility of the work that we publish. This form provides structure for consistency and transparency in reporting. For further information on Nature Portfolio policies, see our Editorial Policies and the Editorial Policy Checklist.

Statistics

For all statistical analyses, confirm that the following items are present in the figure legend, table legend, main text, or Methods section.

n/a	Confirmed	
☐	☒	The exact sample size (*n*) for each experimental group/condition, given as a discrete number and unit of measurement
☐	☒	A statement on whether measurements were taken from distinct samples or whether the same sample was measured repeatedly
☐	☒	The statistical test(s) used AND whether they are one- or two-sided<br>*Only common tests should be described solely by name; describe more complex techniques in the Methods section.*
☐	☒	A description of all covariates tested
☐	☒	A description of any assumptions or corrections, such as tests of normality and adjustment for multiple comparisons
☐	☒	A full description of the statistical parameters including central tendency (e.g. means) or other basic estimates (e.g. regression coefficient) AND variation (e.g. standard deviation) or associated estimates of uncertainty (e.g. confidence intervals)
☐	☒	For null hypothesis testing, the test statistic (e.g. *F*, *t*, *r*) with confidence intervals, effect sizes, degrees of freedom and *P* value noted<br>*Give P values as exact values whenever suitable.*
☐	☒	For Bayesian analysis, information on the choice of priors and Markov chain Monte Carlo settings
☒	☐	For hierarchical and complex designs, identification of the appropriate level for tests and full reporting of outcomes
☒	☐	Estimates of effect sizes (e.g. Cohen's *d*, Pearson's *r*), indicating how they were calculated

Our web collection on statistics for biologists contains articles on many of the points above.

Software and code

Policy information about availability of computer code

Data collection	We used an acquisition device (Dataquest A.R.T., Data Sciences International) for all polysomnographic and actimetry data.
Data analysis	We used a software Neuroscore, Data Sciences International for sleep scoring.<br>We used an open source code from spike2; SleepSpindle 04.s2s for the spindles detection and duration.<br>We used MatLab for the sleep transition analysis.

For manuscripts utilizing custom algorithms or software that are central to the research but not yet described in published literature, software must be made available to editors and reviewers. We strongly encourage code deposition in a community repository (e.g. GitHub). See the Nature Portfolio guidelines for submitting code & software for further information.

Data

Policy information about availability of data

All manuscripts must include a data availability statement. This statement should provide the following information, where applicable:

- Accession codes, unique identifiers, or web links for publicly available datasets
- A description of any restrictions on data availability
- For clinical datasets or third party data, please ensure that the statement adheres to our policy

All data are available within the article, or available from the corresponding authors on reasonable request.

Human research participants

Policy information about studies involving human research participants and Sex and Gender in Research.

Reporting on sex and gender	*Use the terms sex (biological attribute) and gender (shaped by social and cultural circumstances) carefully in order to avoid confusing both terms. Indicate if findings apply to only one sex or gender; describe whether sex and gender were considered in study design whether sex and/or gender was determined based on self-reporting or assigned and methods used. Provide in the source data disaggregated sex and gender data where this information has been collected, and consent has been obtained for sharing of individual-level data; provide overall numbers in this Reporting Summary. Please state if this information has not been collected. Report sex- and gender-based analyses where performed, justify reasons for lack of sex- and gender-based analysis.*
Population characteristics	*Describe the covariate-relevant population characteristics of the human research participants (e.g. age, genotypic information, past and current diagnosis and treatment categories). If you filled out the behavioural & social sciences study design questions and have nothing to add here, write "See above."*
Recruitment	*Describe how participants were recruited. Outline any potential self-selection bias or other biases that may be present and how these are likely to impact results.*
Ethics oversight	*Identify the organization(s) that approved the study protocol.*

Note that full information on the approval of the study protocol must also be provided in the manuscript.

Field-specific reporting

Please select the one below that is the best fit for your research. If you are not sure, read the appropriate sections before making your selection.

☒ Life sciences ☐ Behavioural & social sciences ☐ Ecological, evolutionary & environmental sciences

For a reference copy of the document with all sections, see nature.com/documents/nr-reporting-summary-flat.pdf

Life sciences study design

All studies must disclose on these points even when the disclosure is negative.

Sample size	This study is based on case report observations performed on two animals, non-human primates. We did not calculate the sample-size required because of the case report format.
Data exclusions	We did not need to exclude any data, all collected data are presented in this manuscript.
Replication	mMSLT experiments were replicated for each animal a minimum of 10 times in each condition, and the long-term 12h recordings were replicated 10 times as well.
Randomization	Randomization is difficult to apply to our study, however we have applied randomization in the design of our study in the choice of stimulation parameters during mMSLT between a control experiment, low frequency stimulation or high frequency stimulation while paying attention to have one of each per week.
Blinding	The sleep scoring was performed by an observer in a blind manner, without knowing the OFF-stimulation or ON-stimulation conditions used.

Reporting for specific materials, systems and methods

We require information from authors about some types of materials, experimental systems and methods used in many studies. Here, indicate whether each material, system or method listed is relevant to your study. If you are not sure if a list item applies to your research, read the appropriate section before selecting a response.

Materials & experimental systems

n/a	Involved in the study
☐	☒ Antibodies
☒	☐ Eukaryotic cell lines
☒	☐ Palaeontology and archaeology
☐	☒ Animals and other organisms
☒	☐ Clinical data
☒	☐ Dual use research of concern

Methods

n/a	Involved in the study
☒	☐ ChIP-seq
☒	☐ Flow cytometry
☒	☐ MRI-based neuroimaging

Antibodies

Antibodies used	Primary antibody, rabbit anti-TH (AB152, Sigma-Merck, France; 1:1000)
Validation	We tested this antibody at several dilutions to get the best result. This experiment gives us an opinion on the characteristic dopaminergic degeneration after MPTP treatment and we did not do quantitative analysis, only qualitative.

Animals and other research organisms

Policy information about studies involving animals; ARRIVE guidelines recommended for reporting animal research, and Sex and Gender in Research

Laboratory animals	The study was performed on two adult male NHPs (Macaca fascicularis - CRP Port Louis, Mauritius), 8-10 kg and both 10 years of age
Wild animals	The study did not involved wild animals.
Reporting on sex	Data collected in this study were only from male animals. There is no scientific reason for this, but ethical reasons about limiting the use of animals are partly responsible.
Field-collected samples	Animals were kept under controlled conditions, 12-hour light/dark cycles [light off at 19:00 h], 23±2°C, and 50±5% humidity. Animals were pair housed with other NHP, had access ad libitum to food and water and supplemental fresh fruit and vegetable was given once a day. End-protocol: animals were deeply anesthetized with ketamine and pentobarbital (10mg/kg and 25 mg/kg i.m.), then transcardially perfused with 0.9% saline solution followed by 4% paraformaldehyde in 0.1M phosphate buffer (PB), pH 7.4.
Ethics oversight	In accordance with the policy of Grenoble Alpes University and the Grenoble Institut of Neurosciences (B3851610008) and with French legislation, experiments were performed in compliance with the European Community Council Directive of 2010 (2010/63/UE) for care of laboratory animals. All procedures were reviewed and validated by the “Comité éthique du GIN n̊004” and was authorized by the Direction Départementale des Services Vétérinaires de l’Isère – Ministère de l’Agriculture et de la Pêche, France.

Note that full information on the approval of the study protocol must also be provided in the manuscript.
